# Novel Approaches for Early Detection of Retinal Diseases Using Artificial Intelligence

**DOI:** 10.3390/jpm14070690

**Published:** 2024-06-26

**Authors:** Francesco Saverio Sorrentino, Lorenzo Gardini, Luigi Fontana, Mutali Musa, Andrea Gabai, Antonino Maniaci, Salvatore Lavalle, Fabiana D’Esposito, Andrea Russo, Antonio Longo, Pier Luigi Surico, Caterina Gagliano, Marco Zeppieri

**Affiliations:** 1 Unit of Ophthalmology, Department of Surgical Sciences, Ospedale Maggiore, 40100 Bologna, Italy; dr.fsorrentino@gmail.com (F.S.S.);; 2 Ophthalmology Unit, Department of Surgical Sciences, Alma Mater Studiorum University of Bologna, IRCCS Azienda Ospedaliero-Universitaria Bologna, 40100 Bologna, Italy; 3 Department of Optometry, University of Benin, Benin City 300238, Edo State, Nigeria; 4 Department of Ophthalmology, Humanitas-San Pio X, 20159 Milan, Italy; 5 Department of Medicine and Surgery, University of Enna “Kore”, Piazza dell’Università, 94100 Enna, Italy; 6 Imperial College Ophthalmic Research Group (ICORG) Unit, Imperial College, 153-173 Marylebone Rd, London NW15QH, UK; 7 Department of Neurosciences, Reproductive Sciences and Dentistry, University of Naples Federico II, Via Pansini 5, 80131 Napoli, Italy; 8 Department of Ophthalmology, University of Catania, 95123 Catania, Italy; 9 Schepens Eye Research Institute of Mass Eye and Ear, Harvard Medical School, Boston, MA 02114, USA; 10 Department of Ophthalmology, Campus Bio-Medico University, 00128 Rome, Italy; 11 Eye Clinic, Catania University, San Marco Hospital, Viale Carlo Azeglio Ciampi, 95121 Catania, Italy; 12 Department of Ophthalmology, University Hospital of Udine, 33100 Udine, Italy

**Keywords:** macular edema, artificial intelligence, machine learning, deep learning, retinal imaging, retinopathy, maculopathy

## Abstract

Background: An increasing amount of people are globally affected by retinal diseases, such as diabetes, vascular occlusions, maculopathy, alterations of systemic circulation, and metabolic syndrome. Aim: This review will discuss novel technologies in and potential approaches to the detection and diagnosis of retinal diseases with the support of cutting-edge machines and artificial intelligence (AI). Methods: The demand for retinal diagnostic imaging exams has increased, but the number of eye physicians or technicians is too little to meet the request. Thus, algorithms based on AI have been used, representing valid support for early detection and helping doctors to give diagnoses and make differential diagnosis. AI helps patients living far from hub centers to have tests and quick initial diagnosis, allowing them not to waste time in movements and waiting time for medical reply. Results: Highly automated systems for screening, early diagnosis, grading and tailored therapy will facilitate the care of people, even in remote lands or countries. Conclusion: A potential massive and extensive use of AI might optimize the automated detection of tiny retinal alterations, allowing eye doctors to perform their best clinical assistance and to set the best options for the treatment of retinal diseases.

## 1. Introduction

Retinal diseases such as diabetic retinopathy (DR), age-related macular degeneration (AMD), and others are major causes of vision impairment affecting millions worldwide, particularly those over 50 [1]. Detecting these diseases early is crucial to prevent vision loss. However, there is a shortage of ophthalmologists, especially in developing countries, and eye care services are often focused in advanced hospitals, making access difficult. In recent years, algorithms and software utilizing artificial intelligence (AI) have emerged as valuable tools for early detection, aiding doctors in diagnosis and facilitating differential diagnosis. AI is particularly crucial for remote or isolated communities, as it enables patients to undergo tests and receive quick initial diagnoses without the need for extensive travel and long waiting times for medical consultations and with no need to have physicians present [2]. This review explores innovative technologies and potential approaches for detecting and diagnosing retinal diseases with the assistance of state-of-the-art machinery supported by AI. 

The widespread use of AI has the potential to optimize the automated detection of subtle retinal changes, enabling eye care professionals to provide optimal clinical care and select the most effective treatment options for retinal diseases. Integrating eye care into primary health care is essential for convenient screening and referral. The multilayered retinal tissue is ideal to scan and readily accessible by multi-imaging techniques with the assistance of AI advanced technology. The variability and progression of retinal diseases necessitates several consultations, constant monitoring, and personalized follow-up. AI allows us to support patient management by efficiently analyzing large data, facilitating early diagnosis and improving long-term prognosis [3].

## 2. Digital Health for Current Standard of Care

### 2.1. Telemedicine

Telemedicine offers solutions by providing remote eye care services, particularly important during the COVID-19 pandemic, reducing unnecessary visits to crowded hospitals. Telemedicine applications, aided by advancements in communication networks and artificial intelligence (AI) analysis, have shown promise in screening for retinal diseases like DR in primary care settings, reducing unnecessary consultations [4]. AI-based systems analyzing fundus photographs have demonstrated high sensitivity and specificity in real-world settings. Optical coherence tomography (OCT) provides detailed retinal images and is increasingly used for diagnosing retinal diseases. While AI has been applied to OCT images in research, its real-world application for retinal disease screening has not been extensively studied. However, these platforms often lack the capability to detect various other common retinal diseases. Recent attention has focused on developing AI algorithms for automated detection of such diseases from OCT images. For example, in their study, Liu et al. integrated AI models for detecting retinal pathologies into a telemedicine platform and deployed it in primary care stations in Shanghai, a city with a significant aging population and a shortage of ophthalmologists [2]. Over a three-month trial, the platform screened participants over 50 years old, identifying those with retinal pathologies and referring urgent cases to superior hospitals.

The AI models demonstrated high accuracy in referral decisions, effectively identifying urgent cases with sight-threatening conditions. While some pathologies were missed or falsely detected, overall, the AI models reliably identified retinal disease cases. The platform significantly reduced the workload of remote eye care personnel by filtering out normal cases and facilitating online medical consultations for pathologic cases. In addition to AI performance, the platform’s effectiveness in real-world implementation was emphasized. Online consultations were conducted rapidly, with most urgent cases visiting superior hospitals for further diagnosis. The project increased awareness of retinal conditions among participants and efficiently utilized mobile telecommunication for medical advice delivery. Overall, the OCT-AI–based platform proved valuable for screening retinal diseases in the elderly population, showcasing its potential in remote eye care services [5].

### 2.2. Deep Learning and Machine Learning

Machine learning (ML), created by Arthur Samuel in 1959, is a field of AI where a program exposed to a huge amount of data can learn to recognize specific patterns within those data. This is achieved with the help of multiple interconnected algorithms layered together, each working on recognizing particular features. Collectively, this system is referred to as a neural network, since it attempts to simulate the functioning of neurons in the human brain [6]. Deep learning (DL) is a subdivision of machine learning where multiple artificial neural networks (ANNs) are layered together to better mimic the human brain’s processing capabilities. Convolutional neural networks are a type of ANN, which are widely used for image and video analysis. Successful data interpretation by these programs can be reported in terms of sensitivity, specificity or a receiver operating characteristic (ROC) curve, which plots the true positive rate against the false positive rate. The past two decades have witnessed a boost in AI-powered solutions within the medical field. Digital images and numerical data are frequently used to train AI and ML algorithms.

In the context of the fourth industrial revolution, DL plays a role of paramount importance. Fit to process high-dimensional data without manual feature engineering, DL has demonstrated superior accuracy in various domains such as natural language processing, computer vision, and voice recognition. In medicine and healthcare, DL has primarily been applied to medical imaging analysis, including ocular imaging like fundus photographs and OCT. DL has shown promise in diagnosing various ophthalmic diseases such as DR, glaucoma, AMD, and retinopathy of prematurity (ROP). DL has the potential of screening for ophthalmic conditions, offering a solution to the resource-intensive nature of traditional screening methods [7].

In the case of DR, DL systems have revolutionized diagnostic accuracy, with several studies demonstrating excellent performance in detecting referable DR. Recent advancements have shown DL systems achieving high sensitivity and specificity, even outperforming human experts in some cases. However, challenges remain in translating these results to real-world DR screening programs, particularly in diverse populations and imaging settings [8].

Similarly, DL has shown potential in detecting referable AMD, with studies reporting clinically acceptable diagnostic performance. While some DL systems have been trained and tested on large datasets like AREDS, external validation and generalization across different populations and imaging modalities require further investigation. In the realm of OCT imaging, DL has enabled automated classification of AMD and segmentation of retinal structures with high accuracy. The use of DL frameworks like U-Net has improved boundary and feature-level segmentation, facilitating the identification of pathologies such as choroidal neovascularization and macular edema.

In ROP screening, DL presents a promising solution to address the subjectivity and shortage of trained examiners. Recent studies have demonstrated the efficacy of DL systems in diagnosing plus disease, a critical feature of severe ROP, with high sensitivity and specificity. Automated DL systems could enhance the efficiency and accessibility of ROP screening, particularly in low-resource settings [9].

Overall, DL holds immense potential to revolutionize various aspects of ophthalmic care, from early disease detection to treatment monitoring. However, further validation and integration into clinical practice are needed to ensure the reliability and effectiveness of DL-based approaches in improving patient outcomes. Despite the impressive accuracy of AI-based models in various ophthalmic diseases, several challenges hinder their clinical implementation and real-time deployment in practice. These challenges manifest at different stages, both in research and clinical settings. One significant challenge is the reliance on training data from relatively homogeneous populations, leading to issues with variability in image characteristics and participant ethnicities. Diversifying datasets could help mitigate this challenge. Additionally, there is a scarcity of large datasets for rare diseases and those not routinely imaged, like cataracts, which limits model development. Another concern is the lack of transparency in reporting the power calculation for independent datasets, impacting the robustness of diagnostic performance. Properly performed power calculations are essential to assess algorithm calibration. Furthermore, widespread adoption of AI in healthcare is hindered by concerns about the “black-box” nature of AI systems. Clinicians and patients require transparency regarding how AI algorithms classify diseases. Heat maps highlighting influential image regions may aid interpretability, but challenges remain in their interpretation and dealing with negations. Moreover, current AI screening systems for DR lack stereoscopic qualities, posing challenges in identifying certain lesions. Future AI algorithms incorporating multimodal imaging data may address this limitation. Additionally, variations in medicolegal aspects and regulatory approvals across countries pose hurdles to implementation. Lastly, patient acceptance of AI-based screening varies across populations and settings, influencing its clinical adoption. While some studies report high patient satisfaction, cultural and contextual factors may impact acceptability, posing a challenge to implementation efforts. Addressing these challenges will be crucial for realizing the full potential of AI in ophthalmology and ensuring its integration into clinical practice for improved patient care. In other words, deep learning represents the cutting edge in artificial intelligence and machine learning, ushering in a new era of innovation in the field. In ophthalmology, DL has demonstrated promising diagnostic performance for various retinal diseases, notably diabetic retinopathy and retinopathy of prematurity. Moving forward, it is imperative to conduct further research to assess the clinical feasibility and cost-effectiveness of implementing different DL systems in clinical settings. Addressing the opacity of DL algorithms, known as the ‘black box’ issue, is crucial for enhancing their acceptance among clinicians. Despite the challenges that lie ahead, DL is poised to profoundly impact the practice of medicine and ophthalmology in the foreseeable future [10].

### 2.3. Chat GPT

Chat-Generative Pre-Trained Transformer (ChatGPT) is an AI language model developed by Open AI and could have a potential role in public health. ChatGPT has the ability of generating human-like text based on extensive data which offers chances for supporting individuals and communities to make informed health decisions. ChatGPT could have various applications in community health, such as providing information on public health issues, answering questions about health promotion and disease prevention strategies, explaining the role of community health workers and educators, discussing the impact of social and environmental factors on community health, and offering information about community health programs and services. However, ChatGPT has several limitations in public health, including limited accuracy, biases and limitations of data, lack of context, limited engagement, and no direct interaction with health professionals. Thus, it is important to acknowledge these limitations and use ChatGPT alongside other resources to ensure accurate and effective public health outcomes [11].

### 2.4. Home Monitoring

Home monitoring in healthcare has a long history, traditionally not requiring artificial intelligence. However, recent advancements have incorporated AI; indeed, in modern healthcare, home monitoring attracts substantial interest for detecting disease progression and biomarkers, especially in high-risk populations. It is particularly suitable for diseases requiring rapid intervention or fluctuating conditions, like neovascular AMD, DR or intraocular pressure (IOP) in glaucoma. The potential benefits of home monitoring include increased convenience, decreased costs, fewer infections, more frequent and accurate data collection, and earlier detection and intervention. However, challenges like data acquisition, safe transfer, and integration into healthcare systems do exist. In ophthalmology, chronic conditions like AMD, which often require frequent clinic visits, is well-suited for home monitoring. AMD requires swift detection and treatment. Balancing frequent hospital visits with the risk of late detection is a challenge for patients and physicians. AI approaches are well-suited for home monitoring in healthcare due to several reasons. Firstly, the volume and frequency of data generated by home monitoring may necessitate automation for data processing. Manual grading and analysis by physicians for large amounts of raw data, especially if generated daily or frequently, would be impractical. Secondly, the complexity of some data requires AI processing for meaningful interpretation. AI can perform advanced analyses beyond the capabilities of physicians, such as extracting quantitative information from imaging like OCT scans. Lastly, AI can help compensate for potentially lower imaging or testing quality in home devices compared to clinical devices.

Home monitoring has become increasingly utilized in recent years, especially for the early detection of progression to neovascular AMD [12]. Two FDA-cleared approaches are the ForeseeHome AMD Monitoring System and the myVisionTrack application. The ForeseeHome system, approved by the FDA in 2009, employs a central visual field monitoring device based on hyperacuity. It presents images with artificial distortion to detect metamorphopsia caused by neovascular AMD. The system uses an algorithm to analyze test responses, including a classifier and change detector, to identify progression to neovascular AMD with high accuracy. In clinical trials, the ForeseeHome system demonstrated superior visual outcomes and smaller neovascular lesion sizes compared to standard care. Long-term real-world studies also showed high compliance and predictive value for subsequent progression to neovascular AMD, even in false positive alerts. The myVisionTrack application, on the other hand, utilizes shape discrimination hyperacuity via smartphone or tablet to detect distortion in the central field. While artificial intelligence is not integrated into its algorithm, the application has shown promise in detecting progression to neovascular AMD and is being tested further for its efficacy [13].

Additionally, home OCT imaging has emerged as a valuable tool for monitoring retinal diseases like AMD. Systems like the Notal Home OCT System offer automated quantification of retinal fluid using deep learning algorithms. Prospective studies have shown high agreement between automated analysis and manual grading, enabling detailed characterization of temporal fluid dynamics and potential integration into clinical decision-making.

Other home OCT devices, such as the SELFF-OCT and MIMO02 OCT, are also being developed, with researchers exploring the incorporation of artificial intelligence for automated segmentation and analysis of OCT images. While challenges remain, such as handling large data quantities and ensuring cost effectiveness, home monitoring with AI integration holds promise for enhancing disease management and patient outcomes in retinal diseases [14].

## 3. Artificial Intelligence Applications to Clinics

### 3.1. Diabetic Retinopathy

Diabetic retinopathy (DR) is a major cause of vision loss among working individuals in both developed and developing nations and represents the most severe eye complication of diabetes mellitus (DM). The International Diabetes Federation predicts that by 2040, around 600 million people worldwide will have DM, with approximately one-third developing DR eventually. A meta-analysis of 35 cohort studies involving 22,869 participants revealed a global prevalence of DR at 34.6%, with 10.2% being vision-threatening [15]. This condition contributes to 51% of blindness cases globally. 

Regular screening for DR is crucial to promptly treat and prevent vision loss. However, time and financial constraints pose significant challenges for both eye specialists and diabetes specialists. The efficacy of screening based on fundus photographs is hampered by the limited number of registered eye specialists, particularly those specializing in retinal diseases. Detecting fundus alterations like microaneurysms, hemorrhages, exudates, and neovascularization, DR often requires manual labeling of lesions on fundus images for automated disease screening using ML algorithms. Several DR screening algorithms have undergone prospective studies, offering valuable insights into their performance and clinical applicability. In the US, three FDA-cleared DR screening AI devices—IDx-DR, EyeArt, and AEYE-DS—are classified as moderate to high risk and require premarket approval.

IDx-DR, version 2.0, was the first fully autonomous AI system across all medical fields to receive FDA clearance in 2018. EyeArt and AEYE-DS demonstrated equivalence to IDx-DR for FDA clearance. In the EU, several devices, including EyeArt and IDx-DR, have obtained class IIa approval, indicating a rigorous certification process [16].

The IDx-DR system incorporates multiple biomarker detectors, some utilizing convolutional neural networks. Previous versions were part of the Iowa Detection Program (IDP), featuring algorithms for image quality assessment and detecting various diabetic retinopathy (DR) indicators. IDP demonstrated good performance in diverse populations, including African and Caucasian groups. In a study analyzing images from the Nakuru Eye Study, IDP showed comparable sensitivity and specificity to human graders. 

The IDX-DR system, an improvement over IDP, incorporates deep learning features and achieved enhanced specificity while maintaining high sensitivity. It was validated in a Dutch diabetic care system, showing promising sensitivity and specificity. A recent study conducted by Abramoff et al. enrolled 900 patients and demonstrated IDx-DR’s high sensitivity and specificity in detecting more than mild DR, leading to FDA approval as the first fully autonomous AI diagnostic system [17]. The IDX-DR system is designed to work with the Topcon NW400 non-mydriatic fundus camera and requires four images for analysis. Notably, modifications were necessary to analyze datasets lacking disc-centered images, and the system can handle some image quality issues through the partial overlap of images.

EyeArt, developed by Eyenuk, showed promising results in both UK and US prospective trials, achieving high sensitivity and specificity for detecting DR. EyeArt, developed by Eyenuk Inc., is a Class IIa medical device available in the EU and Canada, but only for investigational use in the US. Similar to other automated DR detection tools, EyeArt automatically excludes poor-quality images and those of the outer eye. It can analyze images from previous encounters to estimate microaneurysm turnover. The system, cloud-based with an application programming interface, facilitates integration into existing imaging and telescreening software. 

Retrospective verification on a database of 78,685 patient encounters showed a screening sensitivity of 91.7% and specificity of 91.5%. In a UK study, it demonstrated sensitivities of 94.7% for any retinopathy, 93.8% for referable retinopathy, and 99.6% for proliferative retinopathy. When tested against the Messidor-2 dataset, it achieved a referable DR screening sensitivity of 93.8% and specificity of 72.2%. In a novel study combining smartphone app-based fundus images with automated AI screening, EyeArt showed sensitivities of 95.8% for any DR, 99.3% for referable DR, and 99.1% for sight-threatening DR, with specificities ranging from 68.8% to 80.4% [18].

AEYE-DS received FDA clearance in 2022 but has not published detailed trial results. Other notable algorithms include SELENA, developed and validated in Singapore and Zambia, respectively, demonstrating high sensitivity and specificity for referable DR detection. Google’s unnamed DR detection system exhibited superior performance compared to regional retina specialists in Thailand, indicating its potential for clinical use. 

Li et al. developed an AI algorithm with high sensitivity and specificity for referable DR detection in Australians, while VoxelCloud Retina and AIDRScreening systems showed promising results in detecting referable DR in Chinese populations. Overall, these algorithms show >85% sensitivity and specificity compared to human graders, with varying requirements for image acquisition and grading standards. However, differences in demographic characteristics and grading standards across studies highlight the need for further validation and standardization in AI-based DR screening. Additionally, higher rates of ungradable images from AI devices may impact clinical workflow efficiency and referral rates, warranting careful consideration in their implementation.

Retmarker, developed in 2011, utilizes a feature-based machine learning approach to detect microaneurysms from color fundus photos, indicating the presence or absence of disease. Implemented in a two-step process for DR screening, it assists in identifying images warranting referral for in-person ophthalmic examination, potentially reducing clinician workload. RetinaLyze, first described in 2003, lacks recent publications reporting its methods or performance on clinical datasets. Although it can identify microaneurysms from fundus photographs, it does not classify images as referable or non-referable for DR. In Europe, it holds a CE mark class I, requiring human oversight and lacking independent certification. Additionally, there are algorithms under development for alternative fundus imaging modalities, such as portable fundus camera photographs and ultrawide field imaging. 

Medios DR, evaluated prospectively, is one example for portable fundus camera photographs, while some algorithms show promise for detecting referable DR using ultrawide field imaging. However, none of these alternative imaging modalities’ algorithms have obtained FDA clearance or CE marking. Several head-to-head validation studies have compared the performance of various AI algorithms for diabetic retinopathy (DR) screening, aiming to understand how these algorithms fare against each other and human graders. However, comparing these algorithms presents challenges due to differences in patient populations and test data sets, which can significantly impact performance. 

Tufail et al. conducted one of the initial head-to-head studies in 2017, comparing EyeArt, Retmarker, and human graders against a third-party reference standard [18]. The study, based on a British population, showed variations in sensitivity and specificity among the algorithms, with human graders outperforming EyeArt and Retmarker in certain aspects. Another study by Grzybowski and Brona compared IDx-DR and RetinaLyze on a small Polish population, highlighting agreement percentages with the reader for DR-positive and DR-negative cases. 

In a larger study by Lee et al. in 2021, five algorithms were evaluated across fundus photographs from VA hospitals in Seattle and Atlanta. Results showed wide variability in model performance, with some algorithms not surpassing VA graders. Importantly, performance varied between cohorts, suggesting sensitivity to demographic and procedural differences. However, these studies have limitations. They often involve limited or homogenous patient populations, hindering generalizability. Moreover, differences in DR management across healthcare systems complicate algorithm evaluation. Lastly, the scarcity of diverse, publicly available data sets underscores the need for additional studies to facilitate meaningful head-to-head comparisons. The cost-effectiveness of implementing AI algorithms for diabetic retinopathy (DR) screening is increasingly significant. 

While AI has shown promise in accurately detecting DR, its cost-effectiveness compared to human graders remains uncertain. Studies on AI screening’s cost-effectiveness yield conflicting results, influenced by factors like geography and deployment strategy. Some studies, especially in high-income countries like the US, suggest that AI screening is more cost-effective due to lower operational costs. However, findings in countries like China and Thailand challenge this, indicating AI screening’s cost-effectiveness even with lower human grader costs. Yet, studies from other regions, such as China and Brazil, suggest AI algorithms may be less cost-effective than human graders. 

Deployment strategy can also impact cost-effectiveness, with semi-autonomous systems showing potential for greater efficiency. Grader costs vary by country, with high-income nations typically facing higher costs, potentially affecting AI device cost-effectiveness [2]. Challenges in estimating cost-effectiveness arise from variations in study periods, complex healthcare systems, and differing impacts on patient outcomes and healthcare efficiency. Integrating AI algorithms into existing billing and reimbursement structures poses significant challenges, especially in countries like the US, lacking standardized billing frameworks for autonomous AI devices. Similarly, other countries face hurdles in adjusting reimbursement policies to accommodate AI technologies effectively. 

Despite recent developments, financial obstacles hinder widespread AI device deployment. To address these challenges, healthcare systems worldwide may need to revise reimbursement policies and develop new billing frameworks tailored to AI technologies. Equity and bias are critical considerations in the deployment of AI algorithms, with concerns surrounding biased outcomes potentially leading to inequitable results. Developers and users of AI devices bear an ethical responsibility to ensure fairness across all communities. Bias can infiltrate AI models during various stages, from data labeling inconsistencies to the exclusion of specific groups from the dataset, potentially resulting in uneven performance across subgroups. 

While various methods exist to mitigate bias during model development, comprehensive evaluation on diverse test cohorts remains the most effective approach. Transparency in model development is essential, with standardized reporting guidelines recommended to detail methodology, training protocols, and evaluations across specific subgroups [3]. Continuous monitoring post-deployment is necessary to assess patient outcomes and ensure equitable access to AI technologies. Regulatory frameworks must evolve to safeguard patient safety, privacy, and autonomy in the era of AI. While the FDA classifies AI algorithms as medical devices, legal challenges persist, particularly regarding liability assignment. 

The “black box” nature of deep learning decision-making presents a hurdle for legal systems, requiring adaptation to address emerging complexities. Internationally, regulatory efforts like the EU’s proposed AI Act and directives aim to establish guidelines for AI device management and liability. However, low- and middle-income countries may lag in regulatory frameworks, potentially widening existing disparities. Future initiatives like the AI-READI project seek to address data set limitations by generating inclusive, high-quality data sets for AI training and validation. Such efforts aim to foster unbiased machine learning models and advance AI applications in healthcare, ultimately enhancing patient care for those with diabetes. In summary, numerous AI technologies are poised to revolutionize DR screening, with some already demonstrating promising performance on prospective datasets. However, bridging substantial knowledge disparities is crucial to drive meaningful advancements in patient care. Few studies directly compare available devices, underscoring the pressing need for further head-to-head validation studies to guide clinicians in selecting appropriate AI solutions. 

While estimation studies suggest potential cost-effectiveness, navigating complex billing requirements and healthcare system variations presents challenges in accurately assessing true costs. Moreover, these algorithms exhibit variable performance across diverse datasets, necessitating ongoing efforts to ensure equitable diagnoses and outcomes upon clinical deployment. Ultimately, while AI devices hold great promise in alleviating the global burden of DR screening, addressing additional knowledge gaps is imperative to harness the full potential of this emerging technology [19].

### 3.2. Age-Related Macular Disease

Accurate AI models have shown promise in supporting clinical management in ophthalmology, particularly in diseases like age-related macular degeneration (AMD), a leading cause of vision loss expected to affect millions globally by 2040. This review aims to provide an overview of recent applications of AI in AMD management and screening, as analyzed in the literature. Some authors conducted a study aiming to understand AMD on a genetic level using machine learning methods. They compared the performance of four techniques—neural network, lasso regression, support vector machine, and random forest—in assessing AMD risk in over 32,000 Caucasian individuals. The analysis also explored the feasibility of predicting AMD risk through genome analysis. 

All models achieved an area under the curve (AUC) of around 0.80 on the same biobank data and approximately 0.70 on a different biobank [20]. Researchers conducted an intriguing study using a deep learning model trained on OCT images to identify biomarkers of delayed rod-mediated dark adaptation (RMDA), a functional biomarker for incipient AMD. The model effectively detected hyporeflective outer retinal bands on macular SD-OCT linked with delayed RMDA, showcasing an acceptable mean absolute error (MAE) [21]. Various algorithms have been developed for the automatic diagnosis of AMD across different imaging modalities. Many of these algorithms focus on segmenting and counting drusen and drusen-like deposits to detect early-stage AMD. 

Yildirim et al. trained a U-Net DL segmenter to identify early AMD OCT biomarkers, achieving high accuracy and potentially aiding in AMD screening by automating patient selection [22]. An investigation introduced an OCT segmenter based on DL that accurately quantified drusen load and improved upon previous methods, demonstrating strong correlation with human readers. It also developed a DL framework to distinguish drusen from reticular pseudodrusen, achieving over 90% accuracy in classification and segmentation [23]. 

Different DL algorithms pretrained for detecting hyperreflective foci, hyporeflective foci within drusen, and subretinal drusenoid deposits from OCT B-scans have been evaluated, achieving an overall accuracy of 87% for identifying early AMD biomarkers. While DL models focusing solely on drusen identification showed good diagnostic performance, the best results were obtained when utilizing multimodal imaging inputs, particularly OCT B scans and OCT angiography (OCTA) or OCT B scans and color fundus imaging [24]. 

A meta-analysis identified the type of AMD and the architecture of the DL model as significant factors affecting diagnostic performance. The ResNet architecture was highlighted as particularly suitable for optimizing AMD diagnosis, with simpler architectures also showing promise in addressing challenges such as vanishing gradients. Recently, the FDA approved iPredict AMD, a DL screening tool capable of detecting referrable AMD with 88% accuracy. Additionally, this tool can predict individual risk scores for the development of late AMD within one to two years [25]. Several studies have demonstrated the effectiveness of DL in segmenting and quantifying subretinal and intraretinal fluid in exudative AMD. Additionally, DL models have been tested for the automatic identification of macular atrophy, a hallmark of advanced AMD. 

Some research showed the high performance of a DL model in identifying six imaging features associated with macular atrophy in AMD patients [26]. Other researchers developed automated algorithms for segmenting retinal pigment epithelial and outer retinal atrophy in dry AMD, achieving results comparable to human graders [27]. Efforts have also been made to assess the risk of progression from early-stage AMD to late-stage AMD, whether neovascular or atrophic. 

Some researchers utilized an ML model combining demographic, genetic, and OCT-based features to predict the risk of conversion to advanced AMD, achieving good results for geographic atrophy (GA) but less reliable predictions for macular neovascularization (MNV) [28]. 

Others trained a DL model on color fundus photographs to recognize AMD stages and then used this information to assess the risk of conversion to neovascular AMD or GA, achieving high accuracy for incident late AMD prediction [29]. In evaluating the risk of conversion to neovascular AMD, some studies proposed a deep sequence approach combining imaging features, demographic, and visual factors with a recursive neural network (RNN) model. This approach showed promising results for predicting exudation in non-exudative AMD eyes over short and long terms, with high generalizability in short-term predictions [30]. The prediction of treatment burden in AMD has been a focus of recent studies. 

Some authors developed a model integrating baseline OCT features, visual acuity, and demographic data to predict the need for intravitreal injections (IVIs) of ranibizumab over a two-year period. Subretinal fluid volume was identified as a key predictor, with around 75% accuracy in classifying low- and high-treatment-requirement subgroups [31]. 

The performance of ML models has been investigated in predicting IVI needs after the loading phase in AMD patients. The SVM model showed the best performance, with an AUC of around 0.80 in predicting few or many injections over two years. Important predictors included fluid in OCT, lesion characteristics, and treatment trajectory in the first three months [32]. 

Also, it has been proposed a probabilistic forecasting model for the number of injections needed over one year, with a mean absolute error of around 2.6 injections per year. In some investigations, the potential of feature learning to predict treatment demand in AMD has been analyzed using a treat and extend regimen, achieving AUCs around 0.80 for both low and high demand [33]. DL technology was also utilized to predict treatment needs. 

Romo-Bucheli et al. developed a DL model combining DenseNet and RNN architectures, achieving good concordance and AUC in predicting low vs. high treatment requirements [34]. Moon et al. presented a DL model in 2023 aimed at guiding treatment choice between aflibercept and ranibizumab based on OCT images. Their attention generative adversarial network (GAN) model outperformed human examiners in predicting anti-VEGF agent-specific short-term treatment outcomes, suggesting potential advantages for clinical practice [35]. ML technology also shows promise in predicting visual outcomes of anti-VEGF treatment. 

The Lasso protocol performed well in predicting visual acuity outcomes, with 5 letters mean absolute error at 3 months and 8 letters at 12 months. Fu et al. achieved even better results using DL technology, particularly through an OCT segmenter providing biomarker quantification and treatment course changes registration [36]. Quantification of GA is crucial for disease monitoring and understanding progression. Balaskas et al. demonstrated the feasibility of residual visual acuity prediction using a random forest model trained with DL-segmented GA biomarkers from OCT images.

Plus, a reverse engineering-based approach has been proposed to identify new potential biomarkers of GA conversion, while a DL method for the automatic prediction of retinal pigment epithelial and outer retinal atrophy progression has been developed. Innovative applications of AI in ophthalmology also include natural language processing models, demonstrating satisfactory responses to medical queries from AMD patients using Chat-GPT. Additionally, You et al. reviewed the promising potential of GAN in AMD imaging for tasks such as conversion, artifact removal, denoising, and database expansion [37].

### 3.3. Vascular Occlusion

Retinal vein occlusion (RVO) ranks as the second most common retinal vascular disease, after diabetic retinopathy [38]. If left untreated, it can cause severe vision loss [39,40]. Studies show that in people aged 30–89, around 0.8% had RVO in 2015, with risk increasing with age and branch retinal vein occlusion (BRVO) being five times more frequent than central retinal vein occlusion (CRVO) [38,41]. Early signs of RVO include retinal hemorrhage, retinal vascular congestion, and cotton-wool spots. If left untreated, RVO can lead to problems like macular oedema and macular ischemia, ischemic optic neuropathy and neovascular glaucoma, vitreous hemorrhage, retinal neovascularization, and tractional retinal detachment, which can cause blindness [42]. Unfortunately, many cases go unnoticed until vision impairment becomes severe, highlighting the importance of instruments for the early diagnosis of this condition.

The retina offers a unique window into the microvasculature due to its direct accessibility for non-invasive examination. This has fueled research interest in the potential of retinal imaging to predict RVO or other cardiovascular events [43,44]. Studies suggest that the fellow eye (the eye not affected by RVO) in patients with RVO may exhibit subtle structural and functional changes despite appearing normal clinically [45,46,47,48]. Recent investigations have shown decreased microvascular density, altered peripapillary microvascular parameters, and thinner lamina cribrosa in both the affected and fellow eyes of RVO patients [49,50,51]. Furthermore, evidence suggests an increased risk of future RVO in the fellow eye compared to the general population [52,53].

While AI has been used to diagnose eye diseases like diabetic retinopathy, macular degeneration, and glaucoma, its use in diagnosing RVO is still limited. This suggests an opportunity for further research in this area. AI-guided analysis of RVOs was studied, focusing on quantitative analysis using OCT and processing qualitative data extracted by color fundus photographs (CFPs) [54,55,56].

Nagasato et al. investigated two AI techniques: DL and support vector machines (SVM). Both were tasked with identifying nonperfused areas (NPA)—regions deprived of blood flow—using OCTA images. The investigation involved 322 OCTA images, half depicting healthy retinas and the other half showcasing retinas with RVO-induced NPA. The DL technique, specifically a deep convolutional neural network (DNN), was trained on these images. The SVM analysis employed a common software library with a specific algorithm. The key metrics evaluated were the accuracy of NPA detection (measured by area under the curve or AUC), sensitivity (ability to correctly identify true positives), and specificity (ability to correctly identify true negatives). Additionally, analysis time was compared between the AI methods and a panel of seven ophthalmologists. DNN emerged as the champion, exhibiting statistically significant superiority over SVM in all assessed parameters. It achieved a near-perfect AUC score (0.986) alongside impressive sensitivity (93.7%) and specificity (97.3%) for NPA detection in RVO-positive OCTA images. While ophthalmologists achieved comparable sensitivity and specificity, their analysis time was significantly longer, averaging over 11 min compared to the DNN’s 3 min. Notably, DNN analysis revealed a focus on the foveal avascular zone, an area often affected by RVO. This study suggests that DL, when coupled with OCTA imaging, holds immense promise for accurately detecting NPA in RVO [57].

Rashno and colleagues also presented an innovative, fully automated method for segmenting and detecting three types of retinal fluid in OCT B-scans. Their method leveraged a combination of graph shortest path algorithms and CNNs to identify sub-retinal fluid, intra-retinal fluid, and pigment epithelium detachment in patients with AMD, RVO, or DR. The investigators reported high accuracy, with an average Dice coefficient exceeding 76% across three major OCT device datasets (Cirrus, Spectralis, Topcon). Additionally, the method effectively segmented fluid in OCT images from the 2017 Retouch challenge, demonstrating its generalization [58].

Although promising, the OCT-based investigation requires a confirmed RVO diagnosis beforehand, relying on OCT angiography for nonperfusion area detection or B-scans for fluid analysis [57,58]. In contrast, research on AI-assisted RVO diagnosis using CFPs remains limited. Anitha et al. explored an AI system for retinal disease classification using retinal images, including RVO [59]. While demonstrating promising sensitivity and specificity, their study was restricted to a fixed set of four diseases, limiting generalization. In a paper from Chen et al. the authors studied the use of AI in CFPs analysis to diagnose RVO. The analysis implied the segmentation of the lesions appearing in CFPs to identify RVO. A panel of ophthalmologists assessed CFP images and assigned one of four classifications: central retinal vein occlusion CRVO, branch retinal vein occlusion BRVO, non-RVO abnormalities, or normal [60]. 

Additionally, four specific lesion types were identified including abnormally dilated and tortuous blood vessels, cotton-wool spots, flame-shaped hemorrhages, and hard exudates. The investigators selected 600 eligible images from 481 patients and merged them with 8000 healthy retina pictures; then, they randomly divided the 8600 images in three subsets in a 2:1:1 proportion: training set, validation set and test set [60]. These images were then analyzed using four prominent CNN architectures for RVO recognition: ResNet-50, Inception-v3, DenseNet-121, and SE-ReNeXt-50 [61,62,63]. All four networks achieved promising results due to their peculiar characteristics. 

While deeper CNNs are generally capable of extracting more complex features, training them can be challenging. Simply stacking convolutional layers does not guarantee improved performance. ResNet-50 addresses this issue by incorporating residual blocks. These blocks allow for direct connections between the first and last layers, facilitating the training process for deeper networks. Inception-v3 utilizes a unique approach with manually designed branches employing various convolution kernel sizes. This design enables the extraction of features at different scales, proving particularly effective in our study for capturing RVO-related lesions of varying sizes. DenseNet-121 builds upon the ResNet architecture by introducing dense connections between every two layers within a block, as opposed to just connecting the first and last layers. 

In CNNs, not all features hold equal importance. SE-ReNeXt-50 incorporates a squeeze-and-excitation block, employing a dedicated branch to learn weights for individual features, effectively addressing this challenge [60]. The outcome demonstrated Inception-v3 to have the best sensitivity and specificity for the identification of both healthy and RVO-affected retina. Similarly, the study compared the performance of four CNN architectures in segmenting retinal lesions such as vascular dilation and tortuosity, hemorrhages, hard exudates, and cotton-wool spots: FCN-32s, DeepLab-v3, DANet, and LesionNet-8s [64,65]. FCN-32s introduced deconvolution layers for upsampling feature maps, establishing a significant milestone in the field of image segmentation allowing to effectively segment objects at various scales [63]. DeepLab-v3 utilizes an encoder–decoder structure incorporating atrous convolution to enable DeepLab-v3 to effectively segment objects with diverse sizes, making it well suited for the task of retinal lesion segmentation [64]. 

The common struggle of CNN architectures with weak relationships between pixels and features within different channels is addressed by DANet, which introduces spatial attention and channel-wise attention mechanisms, improving segmentation accuracy [65]. While the previously mentioned networks were originally designed for natural image segmentation, retinal lesions present unique challenges. LesionNet-8s addresses this by employing a specifically designed architecture suited for segmenting retinal lesions, which typically present unclear boundaries [65]. Among the evaluated CNN architectures, DeepLab-v3 achieved the best results exhibiting a mean sensitivity of 0.74, specificity of 0.97 in retinal lesion segmentation. This method, although utilizing an open CFP dataset and achieving consistent sensitivity and specificity values, is focused on image consistency, and required the investigators to remove identifying information from CFPs [60].

Overall, looking towards the future, AI holds immense potential for early RVO diagnosis and treatment, potentially improving patient outcomes and reducing healthcare resource utilization. This study demonstrates the ability of AI to detect RVO and related lesions within CFPs, paving the way for its future application in clinical settings, particularly in regions with limited access to retinal specialists.

### 3.4. Retinopathy of Prematurity

Retinopathy of prematurity (ROP), a major contributor to childhood blindness globally, presents a diagnostic challenge due to its subclassification by zone, stage, and presence of plus disease. This subclassification suffers from significant intra- and interobserver variability, leading to inconsistencies in diagnosis [66]. Improved neonatal care has led to a rise in the number of premature infants at risk, creating a challenge in determining optimal screening criteria. This challenge lies in balancing the need to identify all severe cases with minimizing unnecessary examinations [67]. 

“Classic” screening methods, particularly relying solely on direct bedside examinations, become less feasible in regions with limited access to trained ophthalmologists. Additionally, inherent subjectivity in clinical ROP diagnosis leads to high interobserver variability, potentially impacting treatment decisions [68,69]. The increasing use of digital fundus photography in ROP documentation and telemedicine programs has paved the way for computer-based image analysis offering the advantage of being immune to fatigue and biases that can influence ROP severity assessment performed by human operators. Recent advancements in AI have shown promise in computer-assisted diagnosis across various medical domains, offering further potential for improved ROP screening. 

AI applied to large-scale image databases of ROP cases can effectively enable an automated, quantifiable, and objective ROP diagnosis, potentially improving screening efficiency and diagnostic accuracy. Therefore, these databases represent a crucial resource for the development and improvement of AI algorithms for automated ROP diagnosis and staging [70,71,72,73]. Notably, the Stanford University Network for Diagnosis of Retinopathy of Prematurity (SUNDROP) trial demonstrated high efficacy with 100% sensitivity, 99.8% specificity, 93.8% positive predictive value, and 100% negative predictive value for detecting treatment-warranted ROP. These technologies are particularly crucial in providing ROP screening and management in regions with limited access to ophthalmologists, as a single provider can screen infants across wider and remote geographic area [74,75,76].

Implementing such methods can also allow non-ophthalmologists, such as technicians or neonatologists, to perform screening photography, reducing reliance on ophthalmologist examinations so balancing high sensitivity (identifying severe cases) with minimal ophthalmologist time investment [77].

While AI-assisted ROP screening holds promise for addressing current limitations, its integration into routine clinical practice is complex. A challenging aspect is the consistency of the AI performance across different real-world settings, presenting variations in camera systems, patient populations, and image quality. Furthermore, the integration of this technology into existing or new clinical workflows is also a critical aspect, requiring careful consideration of user interface design and ensuring minimal disruption to established clinical practices. Beyond these technical considerations, ethical, medico-legal, and regulatory issues also warrant careful attention.

Computer-based ROP diagnosis systems have been in development in the last two decades [78]. Early systems relied on manual feature extraction, such as quantifying vessel dilation and tortuosity, to establish objective severity metrics. However, these methods lacked learning capabilities and relied on pre-determined diagnostic cut-points, resulting in limited agreement with clinical diagnosis [78]. A turning point came with the introduction of machine learning. 

Ataer-Cansizoglu et al. in 2015 utilized a support vector machine (SVM) to analyze traditional features and identify optimal combinations for plus disease diagnosis [79]. While achieving high accuracy (95%), this system required manual vessel segmentation, hindering clinical practicality. The first fully automated approach for plus disease diagnosis emerged in 2017, employing CNNs [9]. Their findings demonstrated the potential of CNNs to match human grader performance, highlighting the need for less human-dependent training data for surpassing human expertise.

Subsequently, Brown et al., in 2018, presented a deep CNN system (i-ROP DL) capable of fully automated three-level plus disease diagnosis [80]. Trained on a large dataset with a single reference standard diagnosis, i-ROP DL achieved an area under the curve (AUC) of 0.98 for plus disease detection. Notably, on an independent dataset, the system demonstrated superior diagnostic agreement with the reference standard compared to most human experts, achieving sensitivities and specificities exceeding 93% for plus disease diagnosis. While most studies have focused on AI for plus disease diagnosis, there is growing exploration of deep learning (DL) for other aspects of ROP assessment. Examples include grading ROP severity, classifying zone or stage specifically, and even aiding in zone identification [81,82]. For instance, the DeepROP DL system demonstrated high accuracy in detecting ROP with a sensitivity of 96.62% and specificity of 99.32% [21,22].

Additionally, Zhao et al. reported a DL system capable of automatically outlining zone 1 on fundus images, potentially serving as a valuable diagnostic tool [83]. Notably, Mulay et al. achieved a groundbreaking application by directly identifying peripheral ROP ridges (stages) within fundus images using DL [84]. These advancements highlight the broad potential of DL for automated and objective ROP diagnosis across various aspects of the disease. However, it is important to note that none of these systems have yet been integrated into routine clinical practice.

CNNs are susceptible to patterns in training data, including both relevant features and confounding factors like image quality, acquisition variations, and pigmentation. If the real-world ROP population differs from the training data (e.g., manufactured images), performance may suffer. Several AI-based ROP approaches have shown promise in research using curated datasets [76]. However, we need to assess their robustness in diverse clinical and technical scenarios.

Real-world image variability depends on the camera system used for the acquisition. Algorithms optimized for one camera system might not perform consistently across different manufacturers. Standardizing imaging devices and regulatory processes for multi-vendor compatibility is crucial (Table 1).

AI-assisted ROP screening has the potential to transform clinical practice, mirroring the success of AI in diabetic retinopathy. Despite implementation challenges, AI presents a transformative future for ROP clinical workflows. AI-assisted screening holds promise for improved detection accuracy and objectivity, offering functionalities like stage diagnosis, pre-plus/severe disease identification, and continuous severity assessment. However, hurdles exist. Integrating AI algorithms with existing equipment (cameras, cloud systems) and validating their performance in diverse populations are initial steps. Beyond basic plus disease detection, AI offers promise for continuous ROP severity scoring, providing a more objective approach. Future directions include heatmap integration to highlight at-risk retinal areas and AI-assisted analysis of OCT images for the early detection of ROP progression.

Telemedicine integration and autonomous AI reading could further streamline the process. Furthermore, AI can play a crucial role in developing evidence-based treatment guidelines and risk stratification by providing objective disease severity assessments. Beyond image interpretation, AI’s potential extends to assisting with image acquisition (landmark identification, autofocus) and labeling for training purposes. The future holds even greater possibilities with the integration of data from new imaging techniques like OCT angiography, potentially leading to a deeper understanding of ROP’s impact on retinal structure [85]. By addressing current hurdles and exploring these possibilities, AI has the potential to revolutionize ROP care and significantly improve outcomes for premature infants.

## 4. New Models for Eye Digital Care

Multiple machine models have been reported to diagnose conditions such as diabetic retinopathy and age-related macular degeneration. However, the development and validation of these technologies are beyond the scope of most eye care professionals [86]. Table 2 below summarizes studies on the validation of AI technology in the sampled publications [87,88,89,90,91,92]. It is important to remember that the clinician may need to use further diagnostic tools in the differential diagnosis, in the confirmation of diagnosis, management and progression of ocular pathologies [93,94,95,96].

## 5. Material and Methods

To collate literature for this review, the authors searched the Pubmed database using the keywords “artificial intelligence detection retinal diseases”. The results generated where then limited to articles from 2022 or newer. The resulting search string was “((“artificial intelligence”[MeSH Terms] OR (“artificial”[All Fields] AND “intelligence”[All Fields]) OR “artificial intelligence”[All Fields]) AND (“detect”[All Fields] OR “detectabilities”[All Fields] OR “detectability”[All Fields] OR “detectable”[All Fields] OR “detectables”[All Fields] OR “detectably”[All Fields] OR “detected”[All Fields] OR “detectible”[All Fields] OR “detecting”[All Fields] OR “detection”[All Fields] OR “detections”[All Fields] OR “detects”[All Fields]) AND (“retinal diseases”[MeSH Terms] OR (“retinal”[All Fields] AND “diseases”[All Fields]) OR “retinal diseases”[All Fields])) AND ((ffrft[Filter]) AND (2022:2024[pdat]))”. A total of 225 article records were therefore retrieved for this study.

## 6. Legal Concerns about Artificial Intelligence and Future Directions

This review actually summarizes the potentiality of AI technologies but it does not deeply analyze specific algorithms currently available. Generically, AI software work likewise combining results from multiple, partially dependent biomarker detectors, analyzing exudates, hemorrhages, neovascularization, cotton wool spots, and abnormal/irregular lesions [97]. Commercially available several AI software for data analysis, utilizing CNNs and giving quality images, provide eye doctors with much data to make early diagnosis of retinal diseases.

AI models bring greater accuracy and efficiency, capitalizing on the skills and interests of patients and physicians [98]. Different strategies of investigation to early diagnosis retinal pathologies could be reliable for patients when eye specialists find the time to explain the AI’s output, how it has technically performed and how the speed of early detection due to AI software might affect the prognosis and bring eventually positive clinical outcomes for the patient.

Despite the numerous expectations, many challenges still remain for worldwide effective application of AI to ophthalmology clinics. The main issue is the huge costs of machinery and network connections. One topic would be choosing hub and spoke locations: high-tech machinery should be placed in hub centers, implementing networking with equipment placed in spoke centers, where just machines for data acquisition should be installed. A low number of physicians should work in hubs, where they analyze quality images and make early diagnoses, whereas technicians and optometrists should work in spokes, where they maintain machines and they perform the acquisition of multi-imaging. This allocation of economical resources would help upper management and the community save money [99].

To date, there are still technical challenges for the clinical implementation and full employment of AI models in real-life clinical practice. Research has been carried out training data sets from rather homogeneous populations, but the reality is different. In fact, AI testing and training with retinal images are often affected by some variants, such as field of view, width of field, image contrast, image magnification, and participant ethnicities. Data set should be diverse according to the variabilities taken into account [100].

In the future, the number of trained ophthalmologists will probably be low, whereas the number of retinal pathologies will increase due to aging of populations, pollution, environmental issues, and unhealthy lifestyles. The capabilities of humans to interact with machines and technology is currently increasing. This phenomenon will further develop since there will be many places, even far from the central hub and near people’s homes throughout each country, where machines will be installed. The powerful interaction between ophthalmologists and ever-improving CNN and DNN algorithms will change healthcare, allowing fast diagnosis and quick detection of ocular pathologies, leading to rapid treatment [101].

Even though AI has shown great promise in improving healthcare and so-called patient-centered care in order to provide early diagnosis, the application in real life of medical AI has created a lot of ethical and privacy concerns [102]. Europe and the World Health Organization have issued global and regional guidelines about the moral impact of medical AI on patients and on global healthcare deployment [103]. Theoretical ethics models are employed to support decision-making processes in applying medical AI to policy, practice and education. Autonomy, beneficence, non-maleficence, and justice are four basic principles for AI applied to medicine. Plus, safety, trust, fairness, privacy, transparency, and responsibility are taken into account in legal regulations and organizational rules in order to allow the widespread use of AI in clinics [104].

AI-based medical software are being developed and released into the market in the recent years, covering many fields of medicine where imaging is of paramount importance. Regulatory authorities, lawyers, and medical experts have been debating medico-legal concerns deriving from the use of AI software tools applied to clinical practice [105]. This aspect is definitely important for software that are trained to provide clinical decision support (CDS). To date, cutting-edge CDS tools do not work in a fully automated way, because the ultimate responsibility for each kind of diagnostic or therapeutic decision is the charge of the physician, who must validate the outcomes of the CDS tool [106].

However, the big issue remains whether AI-based CDS tools used to achieve clinical practice quicker could potentially affect medical malpractice liability. In the advanced technology available, the output might sometimes be wrong in certain cases, which in turn may cause harm and medical malpractice claims to patients. Generally, the physician has to follow the standard of care with available resources [105]. The doctor’s liability may be affected by applying AI tools in clinical practice. There are two possible scenarios: the recommendation provided by AI is correct but the physician chooses not to follow it; the second scenario is that the physician chooses to follow an incorrect suggestion from the AI totally outside the standard of care [107].

It is important to underline that the medical standard of care is constantly changing depending on the state-of-the-art knowledge and available technologies. AI has to be regarded as a useful tool of CDS to confirm medical decision and definitely not as a tool to improve the standard of care by adopting challenging options or artificial decisions.

## 7. Conclusions

The application of AI technology for the early detection of ocular diseases is becoming more and more widespread because it allows physicians to quickly refer to hubs and patients to rapidly have a medical response. However, clinical practice is characterized by several problems of organization and medico-legal issues. A strict law on the application of AI to clinics for medical decisions does not exist yet. Retinal imaging is crucial in eye pathologies and plays a significant role in the diagnosis, grading, and assessment of treatment options. Regardless of fuzzy boundaries and low contrast, AI provides ophthalmologists with an important aid to improve imaging analysis and faster decision-making. Plus, validating a model to early identify eye diseases, such as diabetes, vascular occlusion, ROP, AMD, with multiple imaging modalities (OCT, OCTA, and fundus photography) is highly desirable. Despite some challenges in current clinical practice, AI technology will represent the new trend for easy support of clinical decisions in the future.

## Figures and Tables

**Table 1:** Application of artificial intelligence to retinal diseases.

Disease	AI Devices	AI Tool
DR	IDx-DR, EyeArt, and AEYE-DS	multiple biomarker detectors, utilizing CNN; feature-based C approach to detect microaneurysms from color fundus photos
AMD	U-net DL segmenter, DL framework, ResNet, iPredict, DenseNet	identify and distinguish early AMD OCT biomarkers; address treatment choice
RVO	SVM, ResNet-50, Inception-v3, DenseNet-121, and SE-ReNeXt-50	identify nonperfused areas and detect foveal avascular zone using CNN and DNN
ROP	SVM, i-ROP, DeepROP	analyze traditional features and identify optimal combinations for plus disease diagnosis, using CNN

DR = diabetic retinopathy; CNN = convolutional neural networks; ML = machine learning; AMD = age-related macular degeneration; DL = deep learning; RVO = retinal vein occlusion; SVM = support vector machine; DNN = deep convolutional neural network; ROP = retinopathy of prematurity.

**Table 2:** Current studies on the validation of artificial intelligence technology.

Author	AI Device/Software	Condition	Dataset Used for Deep Learning	Test Sample	Outcome	Area under Curve (ROC)
Zheng et al. [87]	semi-supervised generative adversarial networks (GANs)	retinal disorders	877 OCT images	107,912 OCT images	semi-supervised GANs performed better than the reference supervised DL model	0.99
Adithya et al. [88]	offline deep learning algorithm (DLA)	vitreoretinal abnormalities (VRA)	4319 ocular ultrasound images	421 ocular ultrasound images	DLA showed high sensitivity detecting retinal detachment (97.4%) and choroidal detachment (100%)	0.939
Liu et al. [89]	deep learning system (DLS) for diabetic macular edema	diabetic retinopathy	4295 OCT images	matched images from the same dataset	DLS had 80% specificity and 81% sensitivity, while experienced graders had 59% specificity and 70% sensitivity	DLS scored 0.88, compared with 0.80 for the reference software
Bai et al. [90]	retinopathy of Prematurity AI (ROP.AI)	plus disease in ROP Plus disease in ROP	unspecified as ROP.AI is a proprietary software	8052 retinal images	84% sensitivity, 43% specificity, and 96% negative predictive value	0.75
Wagner et al. [91]	code-free deep learning-based classifiers (CDFL)	plus disease in ROP Plus disease in ROP	retinal images from 6141 neonates	338 retinal images	CFDL models conferred similar performance to senior pediatric ophthalmologists	0.989
Kemp et al. [92]	Medios AI software (FOP NM-10)	referable diabetic retinopathy (RDR)	unspecified as Medios AI is a proprietary software	2327 retinal images	Medios AI compared favorably with an experienced field grader	0.9648

## Data Availability

Not applicable.

## Abbreviations

AI	artificial intelligence
DR	diabetic retinopathy
AMD	age-related macular degeneration
OCT	optical coherence tomography
ML	machine learning
DL	deep learning
ANNs	artificial neural networks
ROP	retinopathy of prematurity
ROC	receiver operating characteristic
AREDS	Age-Related Eye Disease Study
ChatGPT	Chat-Generative Pre-Trained Transformer
IOP	intraocular pressure
FDA	Food and Drug Administration
DM	diabetes mellitus
IDP	Iowa Detection Program
AUC	area under the curve
RMDA	rod-mediated dark adaptation
MAE	mean absolute error
OCTA	OCT angiography
GA	geographic atrophy
MNV	macular neovascularization
RNN	recursive neural network
IVIs	intravitreal injections
RVO	Retinal vein occlusion
BRVO	retinal vein occlusion
CRVO	central retinal vein occlusion
CFPs	color fundus photographs
NPA	nonperfused areas
DNN	deep convolutional neural network
SVM	support vector machines
CDS	clinical decision support
